# Effects of Exercise Intensity and Time on Efficacy of Paclitaxel and Doxorubicin and Immune Microenvironment in the 4T1 Breast Cancer Model

**DOI:** 10.7150/jca.105352

**Published:** 2025-03-31

**Authors:** Liang Zhou, Yonghong Yang, Xiang Luo, Xinling Gan, Chengqi He, Yong Xia, Siyi Zhu

**Affiliations:** 1 Rehabilitation Medicine Center and Institute of Rehabilitation Medicine, West China Hospital, Sichuan University, 610041, Chengdu, China.; 2 Key Laboratory of Rehabilitation Medicine in Sichuan Province, 610041, Chengdu, China.

**Keywords:** exercise, chemotherapy, breast cancer, immune microenvironment

## Abstract

**Background**: Chemotherapy is the mainstay treatment for metastatic triple negative breast cancer (TNBC). However, many patients still die of metastasis after chemotherapy, making it worthwhile to develop strategies to increase chemotherapy efficacy when treating metastatic TNBC. Previous studies have shown that exercise has the potential to inhibit breast cancer metastasis and enhance the effect of chemotherapy, and the level of exercise had a significant effect on tumor metastasis. However, the effect of different doses of exercise-referring to the combination of intensity and duration-on tumor metastasis during breast cancer chemotherapy remains unclear.

**Methods**: 4T1 TNBC subcutaneous tumors were treated with paclitaxel (PTX) and doxorubicin hydrochloride (DOX), as well as different intensities and duration of exercise. Tumor growth, survival, metastatic burden, and the frequencies of some important immune cells were measured to determine the effects, and underlying mechanism, of different exercise doses on the anti-cancer efficacy of PTX and DOX.

**Results**: The combination of high-dose exercise with PTX and DOX promoted metastasis formation, shortened mouse survival, and up-regulated the neutrophil/T lymphocyte ratio in the lungs. In contrast, low-dose exercise synergized with PTX and DOX to suppress metastasis, prolonged the survival of mice, decreased the neutrophil/T lymphocyte ratio, and up-regulated the percentages of NK cells within the metastatic microenvironment. The combination of different exercise dose with PTX and DOX did not affect primary tumor growth.

**Conclusions**: The intensity and time of exercise might affect efficacy of PTX and DOX; however, TNBC patients should be careful concerning the intensity and time of exercises while undergoing chemotherapy. Breast cancer patients might be best served by participating in low levels of exercise, and avoiding excessive exercise, during PTX and DOX therapy.

## Introduction

Breast cancer has surpassed lung cancer as the most common type of malignancy, and its incidence rate is still increasing 1. Most breast cancer-related mortalities result from metastasis to secondary sites, such as the lungs, bone, liver, and brain 2. Neoadjuvant and adjuvant chemotherapy are common treatment strategies for breast cancer 3. Among the cytotoxic agents commonly used to treat breast cancer, paclitaxel (PTX) and doxorubicin hydrochloride (DOX) are commonly used in both neoadjuvant and adjuvant settings. However, some studies showed the pro-metastatic effects of PTX and DOX in mouse models of breast cancer and that this might limit their efficacy 4. Therefore, it is worthwhile to find strategies to increase the efficacy of chemotherapy to combat metastasis in breast cancer.

Exercise has been considered as a pivotal intervention to both prevent cancer before, and to treat cancer after, initial diagnosis. A recent study showed that participation in recommended amounts of exercise (7.5 to 15 metabolic equivalent task [MET] hours per week) was associated with a 6%-10% lower risk of breast cancer 5. Moreover, exercise could enhance the efficacies of cancer treatments after diagnosis 6. Exercise is also a safe and effective adjuvant therapy to reduce cancer-associated side effects 7. Indeed, some reports indicated that exercise after diagnosis might improve the prognosis of early-stage breast cancer patients 8. Exercise has also been shown to impact cancer stem cells through irisin, thereby inhibiting tumor metastasis 9. The parameters of exercise, such as exercise levels and types of exercise, have significant impacts on its effects in cancer patients 8. A narrative review in 2020 reported that high-intensity exercise might promote tumor spread to the body, while moderate-intensity exercise appeared to inhibit cancer metastasis by normalizing tumor vasculature, eliminating circulating cancer cells, and reducing the endothelial cell permeability 10. The FITT principle optimizes exercise outcomes by adjusting frequency, intensity, time, and type. However, little is known concerning how exercise intensity and time affect the efficacy of chemotherapy during breast cancer treatment, including metastasis formation. Therefore, this study specifically focuses on the parameters of intensity and time, aiming to explore their potential impact on the efficacy and mechanisms of chemotherapy.

The effects of exercise on regulating anti-cancer immunity have received significant attention in recent years. Exercise can affect tumors through a variety of mechanisms. Exercise can modulate the properties of tumor cells such as growth and invasion 11. Moreover, exercise can modulate the tumor microenvironment (TME) by regulating neovascularization, influencing the availability of growth factors and nutrients, regulating metabolism, and affecting immune function 6, 12.

The ability of cancer cells to escape immune destruction from T and B lymphocytes, M1-like macrophages and natural killer (NK) cells within the TME is a hallmark of cancer 13. However, accumulation of some innate immune cells within the TME, such as monocytes, tumor-associated neutrophils, and M2-like macrophages can promote tumor progression and metastasis via different mechanisms, such as promoting angiogenesis and inhibiting NK-cell and T-cell cytotoxicity 13. Neutrophils are abundant and heterogeneous leukocytes in peripheral blood, and may suppress cancer growth and metastatic progression by generating anti-cancer factors within the TME 14, 15. However, neutrophils are more frequently reported to facilitate tumor progression through different mechanisms such as promoting cancer cell survival, facilitating angiogenesis, and generating neutrophil extracellular traps (NETs) 15-17. Macrophages are heterogeneous cell types that can be simply divided into M1 and M2 subtypes 18. Classically-activated macrophages can release cytokines and chemokines to foster inflammation leading to anti-cancer activity 18. In contrast, M2 macrophages tend to exert an immuno-suppressive phenotype that promotes cancer progression 18. Several subpopulations of macrophages are resident within lung tissue, namely, alveolar macrophages (AMs) and interstitial macrophages (IMs), both of which can promote lung metastasis progression 19, 20. The immune system plays vital role in cancer development, and exercise has extensively been shown to regulate anti-cancer immunity in preclinical models as well as in cancer patients 6, 21, 22. For example, exercise slowed tumor growth in the 4T1 mammary carcinoma model and these anti-cancer effects are associated with reduction in myeloid-derived suppressor cells (MDSCs) and a relative increase in NK and CD8^+^ T cell activation 23. Exercise also promotes the infiltration of CD8^+^ T cells into pancreatic tumors through the interleukin (IL)-15/IL-15Rα axis 24. In addition, exercise improved the efficacy of a combination of programmed cell death protein-1 (PD-1) inhibitor and radiotherapy 20.

The effects and the underlying mechanisms of the level of exercise, including the intensity and the duration, on breast cancer metastasis during chemotherapy remain undetermined. Therefore, we examined the potential effects of different exercise doses on primary tumor growth and spontaneous metastasis during PTX and DOX therapy and the role of some important immune cell subtypes in the 4T1 breast cancer model. We found that low-doses of exercise synergized with PTX and DOX to suppress spontaneous lung metastasis by reducing neutrophil infiltration and up-regulating T lymphocytes infiltration in the lung tissue. Upregulating the infiltration of NK cells is also linked to the synergistic effects of exercise. In contrast, combination of high-dose exercise with PTX and DOX promoted metastasis formation, shortened the survival of mice, and up-regulated neutrophil/T lymphocytes ratio (NTR) in lungs.

## Materials and Methods

### Reagents

PTX and DOX were purchased from Innochem (Beijing, China). PTX was dissolved in a solvent containing 10% ethanol, 10% Cremophor EL (Innochem, Beijing, China) and 80% normal saline (n.s.) at a final concentration of 1 mg/mL. DOX was dissolved in n.s. at a final concentration of 0.5 mg/mL. PTX and DOX were administered via intravenous (i.v.) injection at 200 µL per 20g body weight every five days. D-Luciferin potassium salt was purchased from BioVision Inc (Milpitas, CA) and dissolved in n.s. at 1.5 mg/mL. Fifteen min before *in vivo* imaging, the mice were administered with 150 mg/kg D-luciferin by intraperitoneal (i.p.) injection. The antibodies used in flow cytometry analysis were purchased from Biolegend (San Diego, CA). The treadmill was purchased from Anhui Zhenghua Biologic Apparatus Facilities (Model: ZH-PT/5S, Anhui, Hefei, China).

### Cell culture

4T1 cells were cultured in DMEM supplemented with 10% FBS, L-glutamine (2 mmol/L), penicillin (100 U/mL), streptomycin (100 μg/mL) at 37 ℃ in a humidified 5% CO_2_ atmosphere. All experiments were performed with mycoplasma-free cells.

### Mouse model and exercise interventions

**Figure 1:**
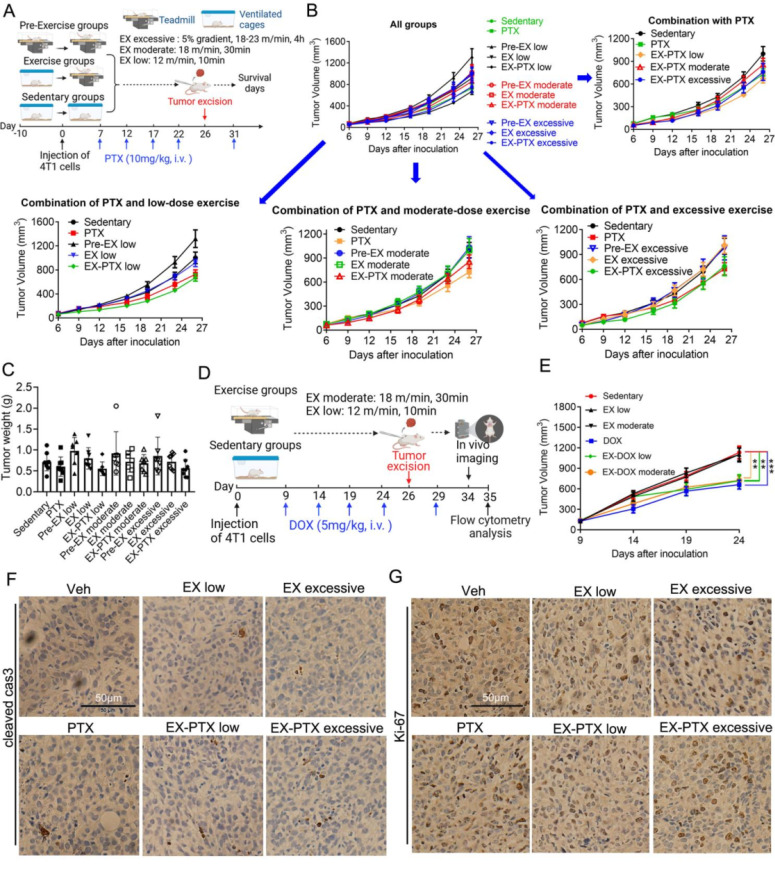
The impact of exercise doses on the efficacy of PTX and DOX in suppressing tumor growth in the 4T1 model. (A) Experimental design of the study using the chemotherapy drug PTX (n = 7-9 mice/group). (B) Tumor volume changes of subcutaneous 4T1 tumors in female BALB/c mice in PTX treatment studies. The data from some selected groups are shown in separate panels to better illustrate the effects of low, moderate, and high-dose exercise on tumor growth. The data are expressed as mean ± SEM. (C) Tumor weight in each group at the time of excision are given. The data were expressed as mean ± SD. (D) Experimental design of the study using DOX as the chemotherapy drug (n = 7-10 mice/group). (E) Tumor volume changes of subcutaneous 4T1 tumors in DOX treatment model. (F, G) The expression of cleaved caspase-3 and Ki-67 in tumor tissue from PTX treatment experiments were analyzed after IHC staining. Representative images from each group are shown. Scale bar, 50 μm.

Ten to twelve-week-old female BALB/c mice were purchased from HFK Bioscience (Beijing, China). All experiments were approved by and performed in strict accordance with the regulations of the Animal Care and Use Committee of Sichuan University. To generate subcutaneous tumors, the mice received a dorsal subcutaneous injection of 2 × 10^5^ luciferase-expressing 4T1 cells. The length and width of the tumor were measured by a vernier caliper. Details regarding the exercise intervention are shown in **Fig. 1A and 1D**.

2.5-5.0 h/week (21-42 min/day) of moderate-intensity activities are recommended by different guidelines to prevent cancer in healthy people and improve physical fitness and quality of life for cancer survivors 7, 25. The most commonly used exercise modalities in preclinical studies include voluntary running, forced treadmill running, and forced swimming 8. We chose forced treadmill running as it can be easily quantitatively controlled, the dose of exercise readily recorded, and running is an economical and highly accessible type of exercise. A previous study indicated that the running speed or relative intensity of exercise could be reflected in the percentage of maximal oxygen consumption (% VO_2 max_ ) measured in mice running on a treadmill 26. Based on several published studies and the above guidelines, the following exercise procedures were applied for mice to achieve different levels of exercise in the present study: low-dose (<50% of %VO_2max_, 12 m/min for 10 min), moderate-dose (about 70% of %VO_2max_, 18 m/min for 30 min), and high-dose (>91% of %VO_2max_, 5% gradient, 18-23 m/min for 4h) 8, 26-28.

To investigate the effects of different duration and intensity of exercise on the tumor growth and survival of the 4T1 model during PTX treatment, mice were divided into different treatment groups as described below and in **Fig. 1A**. After two days of acclimatization to treadmill running for 10 min/day at a speed of 10 m/min, mice were exercised on a treadmill every day during the experiment. For each session of low-dose running, the treadmill speed was set to 12 m/min for 10 min. For each session of moderate-dose running, the treadmill speed was set to 18 m/min for a total of 30 min. For each session of high-dose running, the treadmill speed was set to 18 m/min for the first 30 min. The speed increased 2.5 m/min every 30 min until it reached 23 m/min. For the high-dose exercise protocol, we initially wished for the mice to exercise until exhaustion. However, after searching the literature, we could not find any examples of how long mice took to reach exhaustion on a treadmill, and we found that mice could continue running on the treadmill even after 4 hours of high-dose exercise. Taking into account their daily schedule, we let the mice run for 4 hours as our high-dose exercise protocol.

The mice in each running group were further divided into 3 different subgroups. One subgroup of mice began the exercise 10 days before the tumor cell inoculation and is defined as "Pre-EX group". The second subgroup of mice began the exercise 1 day after the tumor cell inoculation and defined as "EX group". The third subgroup of mice, which is defined as "EX-PTX group", began the exercise 1 day after the inoculation. The mice were treated with PTX by i.v. injection every 5 days.

To investigate the effects of different duration and intensity of exercise on the tumor growth and metastasis during DOX treatment, the mice were divided into different treatment groups as described below and in **Fig. 1D**. After 2 days of acclimatization, the mice exercised on the treadmill every day during the experiment. For each session of low-dose running, the treadmill speed was set to 12 m/min for 10 min. For each session of moderate-dose running, the treadmill speed was set to 18 m/min for a total of 30 min. The mice in each exercise group were further divided into 2 different subgroups. One subgroup of mice began the exercise 1 day after tumor cell inoculation and we defined this as the "EX group". The other subgroup of mice, which we defined as the "EX-DOX group", began the exercise 1 day after tumor cell inoculation and received DOX treatment every 5 days. In both treatment groups, sedentary mice were kept in their home cages during exercise.

### Spontaneous lung metastasis model

4T1 is an aggressive and metastatic TNBC cell line, and lung is a major metastatic site for this model 23, 29. To better simulate cancer metastasis in breast cancer patients after surgery, we removed the primary tumor when the average tumor volume in sedentary mice reached approximately 1000 mm^3^. The primary tumors were resected in a sterile environment as described previously with some modifications 30. Briefly, after the mice were anesthetized with 2% isoflurane, vet ointment was applied to the eyes to prevent dryness. We subsequently sterilized the surgical area surrounding the primary tumor with 70% ethanol and iodophor solution, and then resected the primary tumor with forceps and scissors as described 30. After tumor excision, we closed the excision sites with wound clips which were removed 7 days post-surgery. Mice were kept warm with a heat lamp until fully recovered from the anesthesia. Metastatic growth in the lung and whole body was monitored using an In Vivo Imaging System (PerkinElmer) after i.p. injection of 15mg/kg D-luciferin. The survival of the mice was recorded in the PTX treatment study.

### Flow cytometry analysis

Dissected tumor or lung tissues were cut into small pieces followed by enzymatic digestion with collagenase for 2 h at 37 ℃. The suspensions were filtered through a 70 μm strainer to remove large debris. Red blood cells in the suspension were lysed in Red Blood Cell Lysis Buffer (Biosharp, Hefei, China) for 5 min at room temperature. Following this, cells were stained with the following antibodies for 30 min at 4 ℃ in the dark: PE anti-mouse Ly-6G/Ly-6C (Gr-1), APC anti-mouse CD279 (programmed cell death receptor-1, PD-1), FITC anti-mouse CD4, PE anti-mouse CD8a, FITC anti-mouse/human CD11b, APC anti-mouse F4/80, APC/Cyanine7 anti-mouse CD69, PE/Cyanine7 anti-mouse CD206 (MMR), PerCP/Cyanine5.5 anti-mouse CD45, PE anti-mouse Ly-6G and APC anti-mouse NKp46. After washing with HBSS, cells were analyzed using ACEA NovoCyte (ACEA Biosciences Inc., San Diego, USA). When detecting neutrophils and MDSCs, 100,000 events were analyzed. When detecting T lymphocytes, NK cells, and macrophages, one million events were analyzed.

### Immunohistological analysis of tumor sections

At the end of treatment in the PTX treatment group, subcutaneous tumor tissues were collected and fixed in 4% paraformaldehyde. After embedding in paraffin, the expression of Ki-67, cleaved caspase-3, E-Cadherin and Vimentin in the tumor tissues were analyzed by routine immunohistochemistry (IHC) staining using a DAB detection kit.

### Statistical analysis

Data were represented as means ± SD or means ± SEM and graphs plotted using GraphPad Prism 8.0.1 software. Significant differences between two data sets were evaluated using a 2-tailed Student t test. Analysis for significance was performed by one-way ANOVA to make appropriate multiple comparisons. Survival was analyzed by Kaplan-Meier curves and log-rank tests. Statistically significant p-values were expressed as follows: *p<0.05; **p<0.01; ***p<0.001.

## Results

### The effects of exercise dose on subcutaneous tumor growth in 4T1 breast cancer model during PTX and DOX therapy

We first examined the level of exercise on subcutaneous tumor growth during PTX or DOX treatment. In the PTX treatment group, PTX was dosed at 10 mg/kg once every five days via intravenous i.v. injection. PTX exhibited moderate and non-significant suppression of subcutaneous tumor growth (**Fig. 1B and 1C**). The tumor growth inhibition rate on the day of surgery was 16% (**Fig. 1C**). The level of exercise did not show obvious influences on tumor growth when exercising alone, although low-dose pre-EX slightly promoted the growth of subcutaneous tumor. However, we observed that the level of exercise did not affect the efficacy of PTX treatment on primary tumor growth. Similar results were seen in DOX treatment group of our study (**Fig. 1E**). DOX was dosed at 5 mg/kg once every five days via intravenous i.v. injection. DOX had a significant 42% inhibition of subcutaneous tumor growth 24 days after tumor inoculation. Neither low-dose, nor moderate-dose, exercise influenced primary tumor growth. While DOX inhibited the primary tumor growth, exercise did not affect its efficacy. Consistent with the tumor growth curves, IHC staining of an apoptosis marker (cleaved caspase-3) and a proliferation marker (Ki-67) showed that exercise did not influence the effects of PTX on either apoptosis (**Fig. 1F**) or proliferation (**Fig. 1G**) of cancer cells in vivo.

### The effects of exercise dose on metastasis formation and survival of mice during PTX and DOX therapy

**Figure 2:**
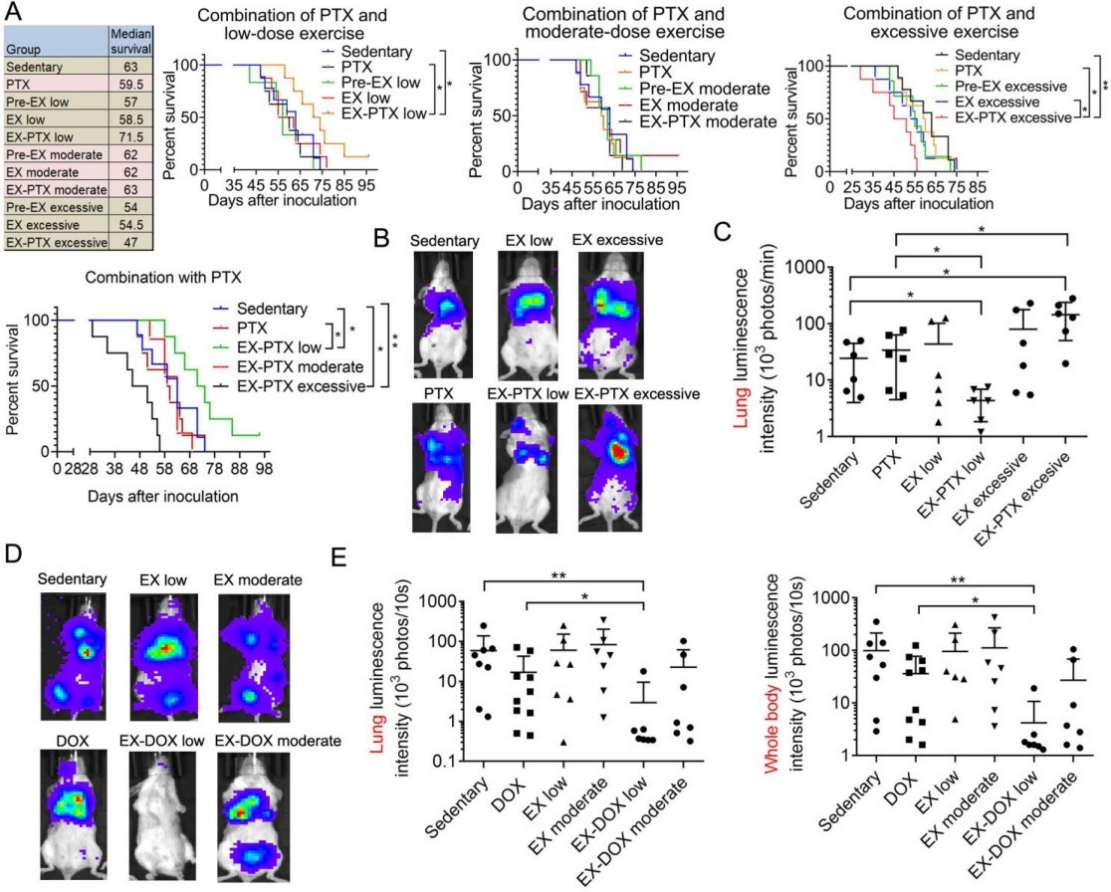
The effect of exercise dose on mouse survival and metastasis in the 4T1 breast cancer model treated with PTX and DOX. (A) The survival data of all 11 experimental mouse groups in the PTX treatment study is shown. The survival curves of selected groups of mice were also shown in separate panels to better illustrate the effects of different exercise doses and PTX. The survival data were analyzed by log-rank test (n = 7-9 mice/group). (B) Representative luminescence images of the mice from each group in PTX treatment studies 29 days after tumor cell inoculation (n = 6 mice/group). (C) Quantification of the luminescence intensity in the lung area in PTX treatment group. (D) Representative luminescence images of the mice from each group in DOX treatment group 34 days after tumor cell inoculation (n = 7-10 mice/group). (E) Quantification of the luminescence intensity in the lung area and the whole body in DOX treatment group. The data were expressed as mean ± SD. **p<0.01, *p<0.05, by one-way ANOVA.

Adjuvant or neoadjuvant chemotherapy is common in the clinical treatment of TNBC 31. However, many TNBC patients still relapse after treatment and succumb to metastatic disease. To mimic the treatment regimen in clinical settings, we excised the primary tumor during PTX and DOX therapy, after which we investigated the formation of metastatic lesions and the survival of the mice. The data showed that PTX caused a nonsignificant decrease in survival of the mice (**Fig. 2A**), which is consistent with a previous finding that PTX might promote breast cancer metastasis in preclinical models 4. While low-dose exercise alone slightly decreased the survival of mice, it synergized with PTX to extend survival (**Fig. 2A**). Moderate-dose exercise did not affect survival time, whether alone or when combined with PTX (**Fig. 2A**). Notably, excessive exercise treatment decreased the survival of the mice, although the results were not statistically significant. The combination of high-dose exercise and PTX resulted in the shortest survival time. We also measured metastasis formation in the mice using *in vivo* imaging. As shown in **Fig. 2B** and** 2C**, exercise alone did not affect lung metastasis formation. However, low-dose exercise synergized with PTX to inhibit lung metastasis formation, whereas the combination of high-dose exercise and PTX accelerated metastatic growth. Similar results were seen in DOX treatment group of our study. Neither low-dose nor moderate-dose exercise alone significantly affected lung metastasis and whole body metastasis formation (**Fig. 2D** and** 2E**). DOX showed modest inhibitory activities on lung metastasis formation. Similarly, low-dose exercise synergized with DOX to markedly decrease metastasis growth. However, the combination of moderate-dose exercise with DOX resulted in a non-significant reduction in lung metastasis formation (**Fig. 2D** and** 2E**).

### The effects of exercise dose on immune cell infiltration in the lung during PTX and DOX therapy

**Figure 3:**
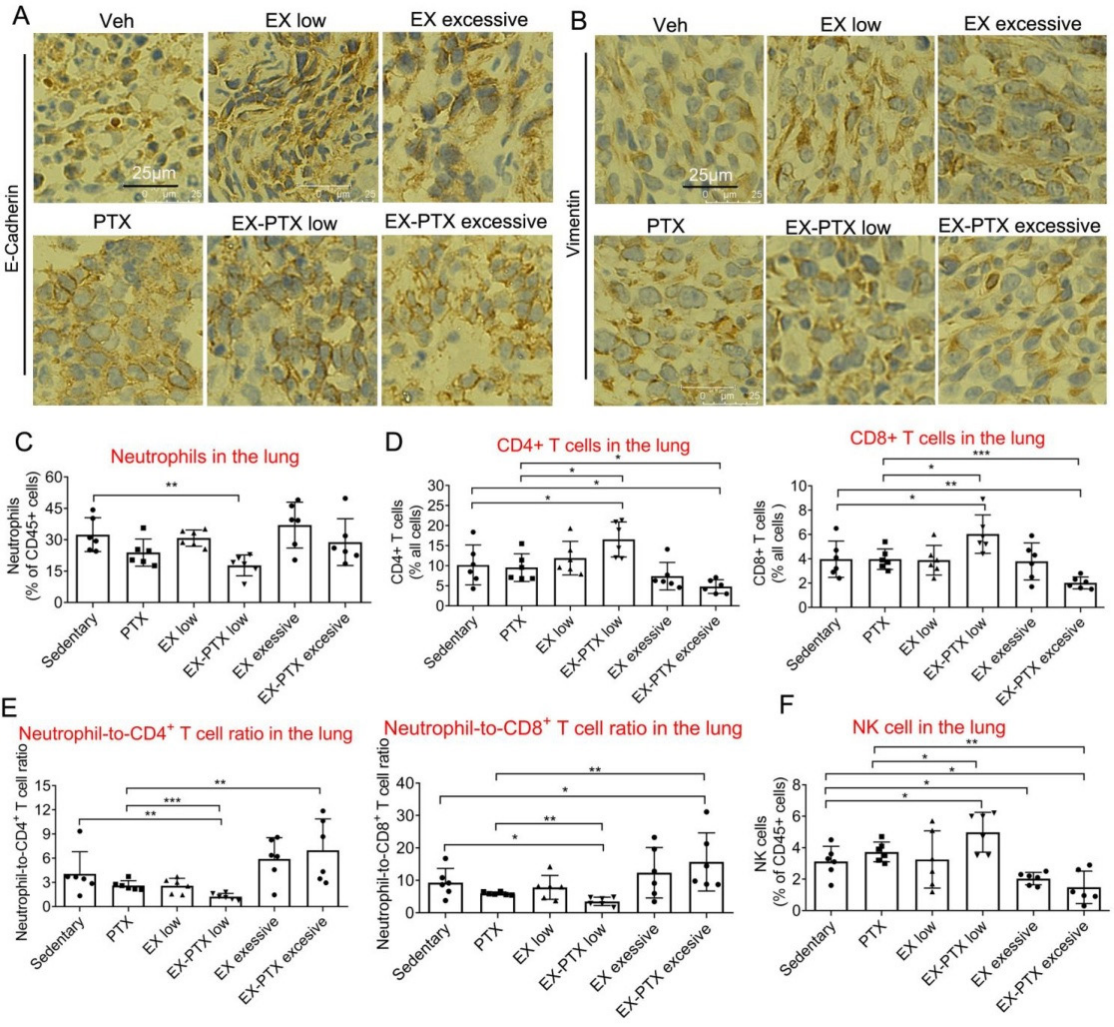
The expression of E-Cadherin and Vimentin in the tumor tissues and frequencies of some important immune cell types in the lung of mice treated with PTX and different doses of exercise training. The expression of E-Cadherin and Vimentin in the tumor tissues in PTX treatment experiments were analyzed by IHC staining. Representative images from each group Are shown in A and B. Scale bar, 25 μm. (C) The effects of different doses of exercise and PTX on the proportion of neutrophils among CD45^+^ leucocytes in the lung after tumor excision. (D) The effects of different doses of exercise and PTX on the percentages of CD4^+^ and CD8^+^ T lymphocytes in the lung after tumor excision. (E) The effects of different doses of exercise and PTX on the NLR in the lung after tumor excision. (F) The effects of different doses of exercise and PTX on the proportion of NK cells among CD45^+^ leucocytes in the lung. n = 6 mice/group. **p<0.01, *p<0.05, by one-way ANOVA.

We questioned whether metastatic growth of 4T1 cell in the lung might be influenced by the properties of the cancer cells and the TME. To address this question, we first explored whether the combination of chemotherapy and exercise affected the metastatic abilities of cancer cells *in vivo*. E-cadherin and Vimentin, two important proteins in epithelial-to-mesenchymal transition (EMT) were detected in the tumor tissue. Data indicated that expression of either protein showed no difference between the groups in PTX treatment study (**Fig. 3A** and** 3B**).

Next, we speculated that the immune TME might be involved in the regulation of lung metastasis caused by combination of exercise and chemotherapy. Neutrophils are thought to possess pro-tumorigenic effects in certain circumstances 16, may promote lung metastasis of breast cancer 32. Previous studies showed that exercise could regulate neutrophil frequency 33, and we measured their presence in both PTX and DOX treatment models. In PTX treatment model, low-dose exercise alone with PTX significantly decreased neutrophil frequency in the sites of metastasis (**Fig. 3C**). T lymphocytes are the main anti-cancer immune cell types in the body and most cancer immunotherapies are based on their tumor cytotoxic effects 34. As shown in **Fig. 3D**, low-dose exercise synergized with PTX to increase the levels of both CD4^+^ and CD8^+^ T lymphocytes while the combination of high-dose exercise and PTX significantly down-regulated these cell types.

A high circulating neutrophil-to-lymphocyte ratio (NLR) is associated with a poor prognosis in breast cancer and other cancer types 16, 35, 36. We found that low-dose exercise synergized with PTX to downregulate NLR while high-dose exercise and PTX significantly increased this ratio (**Fig. 3E**). NK cells are cytotoxic cells within the innate immune system that can eliminate tumor cells without prior immune sensitization 37. They are the most exercise-sensitive immune cell type, exhibiting acute mobilization of the circulation during exercise 38. Previous findings showed that exercise could increase the anti-cancer effects of NK cells through mobilization and tissue redistribution alone, or in combination with other cancer treatment strategies 23, 39, 40. Analysis of NK cell frequency indicated that the combination of low-dose exercise and PTX has the ability to up-regulate the percentage of NK cells within the metastatic site (**Fig. 3F**). High-dose exercise, either alone or combination with PTX, decreased NK cell frequency at metastatic sites within the lung (**Fig. 3F**). This may be because high-dose or prolonged-duration exercise creates favorable conditions for lung metastasis by regulating the release of inflammatory factors or altering the functions of immune cells 41, 42.

**Figure 4:**
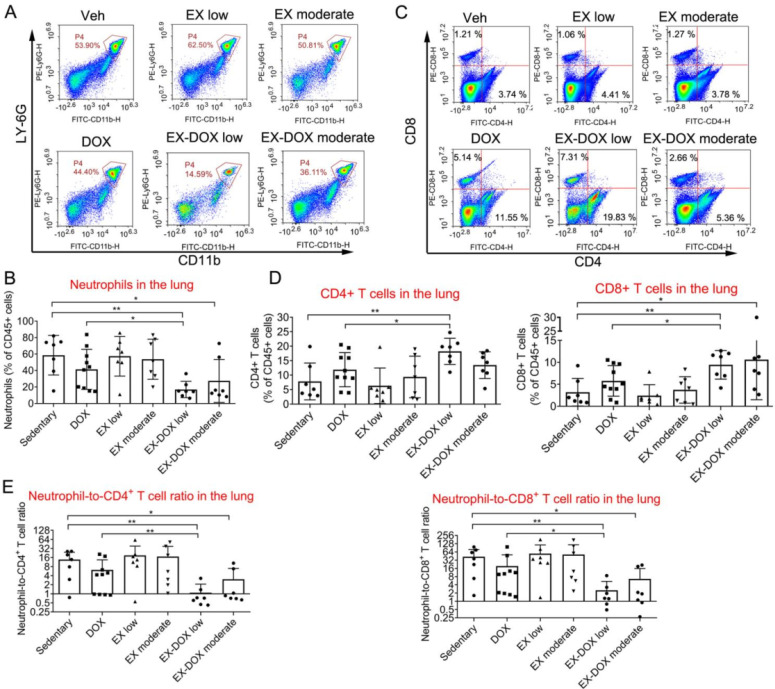
The effects of different doses of exercise training and DOX treatment on the frequencies of some immune cell types in the lung of mice after tumor excision. (A, B) The effects of different doses of exercise and DOX on the proportion of neutrophils among CD45^+^ leucocytes in the lung. Representative flow cytometry panels of lung infiltrating neutrophils cells are shown in A. The quantification of the proportions from each group are shown in B. (C, D) The effects of different exercise doses and DOX on the percentages of CD4^+^ and CD8^+^ T lymphocytes among leucocytes in the lung. Representative flow cytometry panels of lung infiltrating T lymphocytes are shown in C. The quantification of the proportions from each group are shown in D. (E) The effects of different doses of exercise and DOX on the NLR in the lung. The data were expressed as mean ± SD. n = 7-10 mice/group. **p<0.01, *p<0.05, by one-way ANOVA.

Similar results were observed in the DOX treatment groups. Neither DOX nor exercise affected the frequency of neutrophils among CD45^+^ leucocytes in lungs (**Fig. 4A** and** 4B**). However, combination of low-dose exercise and DOX markedly reduced the frequency of neutrophils compared with either vehicle, DOX alone, or low-dose exercise alone. Although the combination of moderate-dose exercise and DOX reduced the frequency of neutrophils, the frequency of neutrophils was higher in this group than that seen in combination with low-dose exercise. Moreover, the combination of low-dose exercise with DOX increased the frequency of both CD4^+^ and CD8^+^ T lymphocytes by at least two-fold when compared with vehicle or low-dose exercise, and more than 50% when compared with DOX treatment alone (**Fig. 4C** and** 4D**).

**Figure 5:**
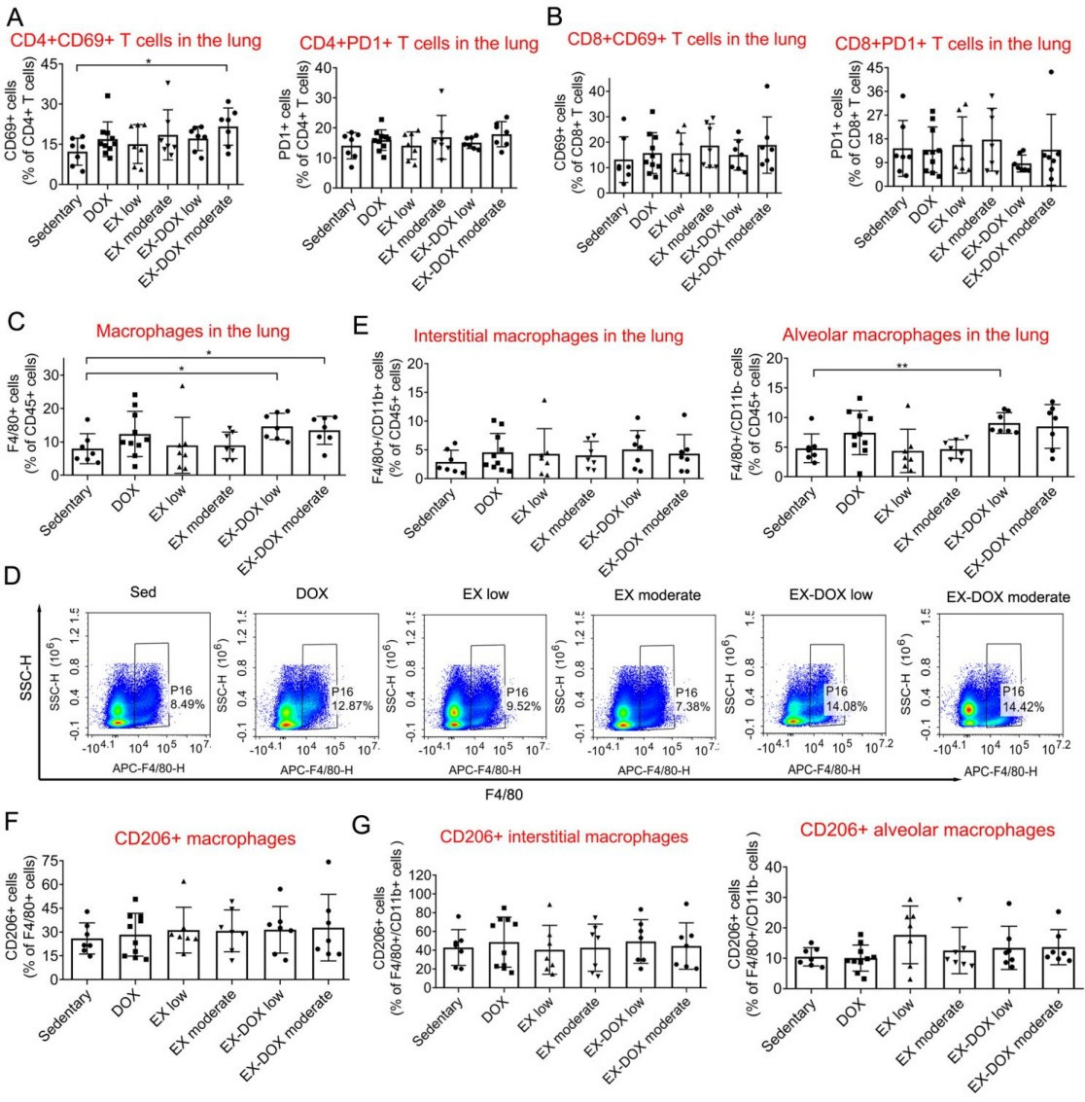
The effects of different doses of exercise and DOX treatment on T cell expression of CD69 and PD-1 and the proportion of macrophages in the lung after tumor excision. The effects of different doses of exercise and DOX on the expression of CD69 and PD-1 on CD4^+^ and CD8^+^ T cells in the lung are shown in A and B, respectively. The effects of different doses of exercise and DOX on the proportions of F4/80^+^ macrophages, F4/80^+^/CD11b^+^ interstitial macrophages (IMs) and F4/80^+^/CD11b^-^ alveolar macrophages (AMs) among CD45^+^ leucocytes in the lung are shown in C-E, respectively. The percentages of CD206^+^ M2 macrophages among F4/80^+^ macrophages, IMs and AMs are shown in F and G, respectively. The data were expressed as mean ± SD. n = 7-10 mice/group. **p<0.01, *p<0.05, by one-way ANOVA.

After analyzing the NLR ratio, we determined that the combination of low-dose exercise with DOX shifted the balance between immune-suppressive and anti-cancer effector cells within the lung microenvironment (**Fig. 4E**). The expression of CD69, a T cell activation marker, and PD-1, an inhibitory receptor regulating T cell exhaustion, were not affected after the treatment including combination intervention (**Fig. 5A** and** 5B**). The proportions of macrophages were also analyzed. The combination of DOX and exercise did not affect the percentages of total macrophages (**Fig. 5C**), AMs, or IMs (**Fig. 5E**). Similarly, expression of CD206, a typical marker of pro-tumor M2 macrophages, did not change as well (**Fig. 5F** and** 5G**). Thus, macrophages are likely not involved in the effects of exercise on regulating metastatic growth during PTX and DOX therapy.

### The effects of moderate-dose exercise on immune cell infiltration in the tumor tissue

**Figure 6:**
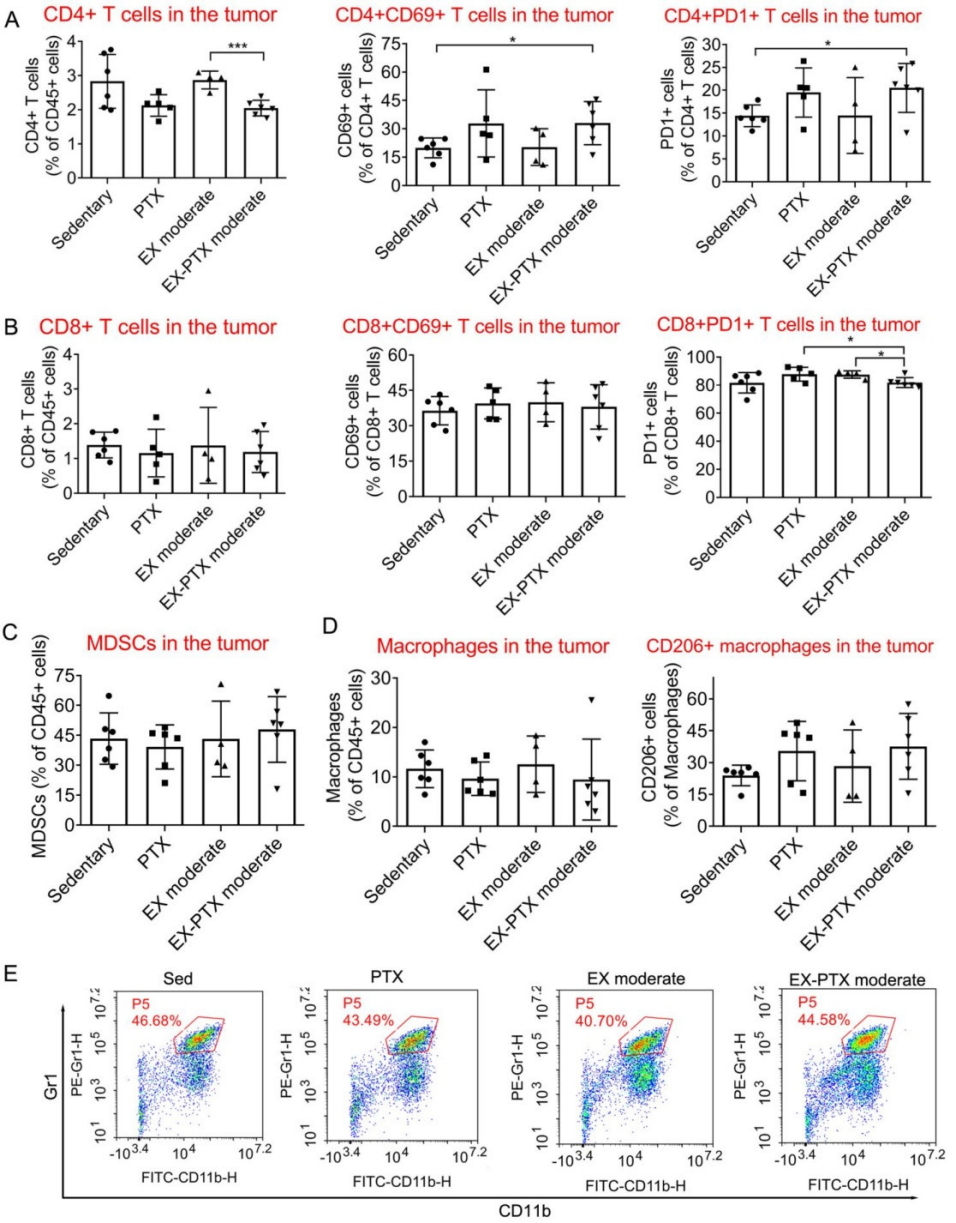
The effects of moderate-dose exercise and PTX treatment on key immune cell populations within the tumor tissue of the 4T1 breast cancer model. One day after the inoculation of 4T1 cells, the mice were divided into 4 groups: sedentary group, PTX treatment group, moderate-dose exercise group (EX), and a combination group (EX-PTX). PTX was administered i.v. every 5 days at 10 mg/kg. The first administration began on day 7 after tumor cell inoculation. One day after the inoculation, the mice began to exercise on a treadmill. For each session of running, the treadmill speed was set to 18 m/min for 30 min/day and the tumors were subsequently collected for flow cytometry analysis. The percentages of CD4^+^ T lymphocytes within the leucocyte population, and the expression of CD69 and PD-1 in CD4^+^ T cells are shown in A. The percentage of CD8^+^ T lymphocytes and the expression of CD69 and PD-1 in CD8^+^ T cells are shown in B. The percentages of MDSCs within the leucocyte population in the tumor are shown in C. The proportions of F4/80^+^/CD11b^+^ macrophages within the leucocyte population and CD206^+^ M2 macrophages in the tumor are shown in D. Representative flow cytometry plot of Gr1+/CD11b+ MDSCs in C. The data were expressed as mean ± SD. n = 4-6 mice/group. ***p<0.001, *p<0.05, by one-way ANOVA.

We were curious as to how the combination of exercise and chemotherapy affects the frequencies of important immune cells within the tumor tissue as our findings indicate that exercise dose does not have significant impacts on the growth of primary tumors during PTX and DOX therapy. To closely study this question, we selected one representative combination strategy to investigate the effects. We analyzed the effects of moderate-dose exercise on the frequency of some immune cells within the microenvironment of subcutaneous 4T1 tumors during PTX treatment. Moderate-dose exercise did not alter the effects of PTX on the proportion of CD4^+^ T lymphocytes (**Fig. 6A**), CD8^+^ T lymphocytes (**Fig. 6B**), myeloid-derived suppressor cells (MDSCs, **Fig. 6C**), CD11b^+^/F4/80^+^ macrophages (**Fig. 6D**) among leukocytes within the tumor tissue. The expression of both CD69 and PD-1 in CD4^+^ and CD8^+^ T lymphocytes (**Fig. 6A** and** 6B**), and CD206 expression in macrophages were also not changed (**Fig. 6D**). In summary, the addition of moderate-dose exercise to PTX did not influence the frequency of the above immune cell types in the tumor tissue.

## Discussion

Anthracycline and taxane-based chemotherapy are mainstay treatments for TNBC 43. Metastasis to other sites of the body is the principal reason for TNBC-related death 43. However, some studies showed the potential risk of promoting metastasis by these chemotherapy drugs 4. The role of exercise in modulating cancer incidence and progression has received attention as exercise can have a critical impact on cancer prevention, treatment outcomes, and regulating the side effects associated with cancer treatment 6. The parameters of exercise, such as intensity, time, and type of exercise, might affect the effects of exercises during cancer metastasis. The role of exercise, and its intensity, on cancer metastasis remains controversial. Some reports showed that exercise has no influence on any markers of cancer metastasis incidence using preclinical models 8, 44. However, a narrative review indicated that high-intensity exercise might promote cancer metastasis while moderate-intensity exercise suppressed cancer metastasis by regulating angiogenesis in the tumor tissue, killing circulating cancer cells, and reducing the permeability of endothelial cells 10. Nevertheless, the effects of exercise during and after chemotherapy on breast cancer metastasis remain unclear 8. Therefore, it is necessary to investigate the effects of different doses of exercise on the treatment outcomes of chemotherapies, including both primary and metastatic tumor growth in breast cancer. To our knowledge, this is the first study to investigate the effects of different exercise doses on metastasis formation during PTX and DOX therapy using the 4T1 mouse metastatic breast cancer model.

Exercise can cause systemic changes to the body including molecules in circulation, signaling pathways, immune regulation, and metabolism 6, 12. Therefore, the mechanistic effects of exercise on cancer metastasis are likely quite complicated. Exercise can regulate angiogenesis, which has a paradoxical role in metastasis 12. Specifically, cancer cells metastasize to other parts of the body through blood vessels 12; however, blood vessels can also transport anti-cancer immune cells and therapeutic drugs to the metastatic site 12. Metabolic reprogramming may be another key aspect of the anti-tumor effect of exercise. Exercise may inhibit tumor cell metastasis by regulating the metabolic levels of distant organs 45. Exercise can alter the concentration of nutrients and growth factors in circulation and within the metastatic TME, exert direct anti-tumor effects, and restore anti-tumor immune responses by altering the acidic, nutrient-depleted TME 13. The regulatory effects of exercise on the immune microenvironment have attracted significant attention as of late 23. To successfully form metastases, cancer cells must evade immune destruction by cytotoxic T lymphocytes (CTLs) and specific subtypes of B lymphocytes, which play key roles in mediating immune surveillance and antitumor immunity, anti-cancer macrophages, and NK cells. In addition, the accumulation of some innate immune cells, such as neutrophils and tumor-promoting macrophages, at metastatic sites can also contribute to the occurrence of metastatic spread 13. Cytotoxic CD8^+^ T cells (CTLs) possess a vital role in restricting tumor growth. CD4^+^ T helper cells can amplify the CTL response against tumor-associated antigens and eliminate malignant cells 46. One mechanism by which tumors evade immune destruction is by up-regulating the expression of PD-1 to impair the proliferative response 13. In this study, combination of low-dose exercise with PTX and DOX therapy significantly increased the proportions of both CD8^+^ and CD4^+^ T cells within the metastatic site when compared with either exercise or chemotherapy alone. The expression of PD-1 in T cells was not influenced after the intervention. Therefore, the addition of immune checkpoint inhibitor in the combination regimen of low-dose exercise and chemotherapy might improve the anti-metastasis effect in breast cancer. This is a promising future direction based on the findings of this study as most preclinical studies need to be conducted before such combination strategies can be translated into clinical trials. CD4^+^/Foxp3^+^ T cells, or regulatory T cells (Tregs), can induce immune tolerance in tumors. A preclinical study showed evidence that exercise exhibited anti-tumor immune efficacies by upregulating the intra-tumoral CD8^+^/Treg cell ratio in breast cancer 47. Therefore, the proportion of Tregs should be investigated to elucidate the anti-metastasis mechanism of the combination treatment in the future. NK cells are a group of innate immune cells that possess pivotal anti-cancer abilities, and are the most responsive immune cell types to physical exercise 38. Previous studies showed that exercise enhanced both the infiltration and activation of NK cells to exert their anti-cancer activities alone, or in combination with PD-1 blockade and focal radiotherapy 23, 29. It is of interest to know the effects of combination chemotherapy and exercise on NK cells and, in the present study, we found that the frequency of NK cells is consistent with the changes of metastasis intensity after combination interventions. This suggests that the mechanisms regulating this effect should be further investigated.

A high circulating NLR portends a poor prognosis in multiple cancer types including breast cancer, and targeting neutrophils has become a promising therapeutic strategy to treat cancer 16, 35, 36. Neutrophils are multifaceted cell types that can be divided into pro-tumor phenotype (N2) and anti-tumor phenotype (N1). Recent studies indicated that neutrophils are frequently thought to facilitate tumor progression and metastasis by modulating cancer cell survival and migration, immune function, and angiogenesis 14. Neutrophils can support metastatic growth by promoting the formation of NETs 15. In the current study, combination of low-dose exercise with PTX and DOX therapy significantly reduced the proportion of neutrophils and NLR. To more accurately elucidate the role of neutrophils in mediating the anti-metastasis effects of the combination, the proportion and functions of both N1 and N2 neutrophil subtypes and the formation of NETs are worthy of further investigation.

Macrophages in the TME are inversely correlated with clinical outcome of cancer patients. They can be divided into pro-tumor phenotype (M2) and anti-tumor phenotype (M1). The M2 subtype is mostly thought to promote cancer progression. Macrophages within the lung can be divided into AMs and IMs; however, we did not observe significant changes in the proportions of total and M2 subtype of macrophages after the combination treatment in either in AMs or in IMs. Therefore, we conclude that macrophages are likely not involved in the regulatory effects of exercise on metastasis during PTX and DOX therapy.

This study has some limitations, and we will discuss them as well as future directions. We used 4T1 xenograft model in this study. Orthotopic and spontaneous breast cancer models would be good alternative choices as such models permit the immune response to be examined within the mammary gland, and the conclusions drawn may be more convincing. In future studies, we will endeavor to use these types of breast cancer models, such as the MMTV-PyMT mouse model, other transgenic breast cancer models, and PDX models.

The exercise regimen used in this study contains three different parameters, but the intensity and time are different for each. Four hours of high-intensity exercise a day in the high-dose exercise protocol might be too long to be a realistic model. In future studies, we will explore the effects of exercise with more parameters, such as intensity and time, on the efficacy of breast cancer chemotherapy to explore the importance of intensity and time, especially for low-dose and excessive exercise.

We found that low-dose exercise can synergize with PTX and DOX therapy to increase the number of T lymphocytes. Immunotherapy that achieves good efficacy in a variety of tumor types relies on restoring T cell function. Therefore, combining chemotherapy and immunotherapy with low-dose exercise may achieve better anti-cancer effects. We did not conduct an experiment that addresses this issue in the present study. In our current study, we observed differences in the effects of high-intensity and long-duration exercise compared to low-intensity and short-duration exercise when combined with doxorubicin or paclitaxel. However, the underlying mechanisms remain unclear based on the available data. One possible explanation is that varying exercise intensities lead to differential activation of the immune system 21. For instance, high-intensity exercise can induce a stronger immune response but may also inhibit CD8^+^ T cell activation and trafficking, potentially resulting in immunosuppression or immune evasion 48. Additionally, the distinct regulatory effects of different exercise intensities on metabolism may contribute to these discrepancies 49. For example, the accumulation of lactate within tumors could influence tumor cell survival 50. The impact of exercise on inflammation may also affect tumor growth and metastasis. High-intensity exercise might promote tumor progression by activating robust inflammatory pathways 51, particularly through macrophages 52, which could create a favorable environment for tumor cell proliferation. In future studies, we will explore the anti-cancer effects of low-dose exercise, chemotherapy, and immunotherapy when appropriate exercise doses are confirmed in more breast cancer models.

## Conclusions

Taken together, we determined that exercise dose did not influence the effects of PTX and DOX to suppress primary tumor growth in the 4T1 breast cancer model. Importantly, the data indicated combination of high-dose exercise and PTX and DOX therapy promoted metastasis formation and shortened the survival of mice, whereas low-dose exercise synergized with PTX and DOX therapy to markedly suppress spontaneous metastasis growth and prolong survival. Combination of low-dose exercise and PTX and DOX therapy could increase the proportion of T lymphocytes while decreasing that of neutrophils within the lung, which might contribute to anti-metastatic effects. In contrast, the combination of excessive exercise and chemotherapy up-regulated NLR at the sites of metastasis. NK cells might also be involved in regulating the growth of metastasis during combination treatment. However, the underlying mechanism of how combination of different levels of exercise and chemotherapy regulates the frequencies of the immune cells requires further investigation. Overall, this study revealed that breast cancer patients might benefit from participating in low-level exercise, as well as avoiding excessive exercise, while undergoing treatment with either PTX or DOX.

## Abbreviations

TNBC: triple negative breast cancer
PTX: paclitaxel
DOX: doxorubicin hydrochloride
MET: metabolic equivalent task
TME: tumor microenvironment
NK: natural killer
AMs: alveolar macrophages
IMs: interstitial macrophages
MDSCs: myeloid-derived suppressor cells
PD-1: programmed cell death protein-1
NTR: neutrophil/T lymphocytes ratio
EMT: epithelial-to-mesenchymal transition
NLR: eutrophil-to-lymphocyte ratio
EX: exercise group
IGF-1: insulin-like growth factor
